# Safety, tolerability and feasibility of adenosine stress CMR in high-risk patients

**DOI:** 10.1186/1532-429X-17-S1-P124

**Published:** 2015-02-03

**Authors:** Amardeep Ghosh Dastidar, Alexander Carpenter, Catherine R Wilson, Nauman Ahmed, Chris B Lawton, Mark Hamilton, Chiara Bucciarelli-Ducci

**Affiliations:** NIHR Cardiovascular Biomedical Research Unit, Bristol Heart Institute, Bristol, UK

## Background

Adenosine stress cardiovascular magnetic resonance (CMR) provides effective cardiac prognostication in patients with suspected coronary artery disease. However its use has been limited in high-risk patients and some reservations exist about offering adenosine stress CMR in patients with significant aortic stenosis, asthma, severe left ventricular (LV) systolic dysfunction, significant left main stem (LMS) disease and age >80years.

## Aims

To determine the safety, tolerability and feasibility of stress CMR, in the diagnosis of inducible myocardial ischaemia in high-risk individuals as compared to non-high risk.

## Methods

From April 2013-February 2014, 542 consecutive adenosine stress-CMR examinations were performed. Cardiovascular and symptomatic response to adenosine stress was recorded in every patient. The CMR protocol included cine (long and short axis), stress and rest first-pass perfusion imaging, and late gadolinium enhancement (LGE) imaging. Patients were classed as high-risk if any of the following criteria were fulfilled: age >80yrs, asthma or chronic obstructive pulmonary disease (COPD), significant left main stem stenosis and moderate-severe or severe aortic stenosis or severe LV systolic dysfunction (ejection fraction <40%). Comparisons were made using the Fisher exact test for binary variables.

## Results

542 consecutive stress-CMR were included in the analysis (mean age 64 years and 71% males), out of which 99 patients (18%) met the criteria for high-risk. Overall, the complete stress-CMR protocol was successfully performed in 93% of patients. The high-risk group had a drop out rate of 1% compared to a rate of 8% for the non-high risk group (p=0.01). Adequate stress response (symptomatic and/or cardiovascular) was achieved in 98% of high-risk and 99% of non-high risk (p=0.62). 74% of high-risk patients received incremental increase in adenosine dose. Overall, no serious adverse events were noted. When compared to non-high risk, the high-risk group more commonly presented with inducible perfusion defects (63% vs 39%, p=0.0003) and LGE (74% vs 50%, p=<0.0001)(Table 1).

**Table 1:** Table 1

	Total (542)	High-risk (99)	Non-high risk (443)	P-value
Male (%)	387 (71.4)	70 (70.7)	317 (71.6)	0.90
Stress response	535	97	438	0.62
Drop out	35	1	34	0.01
Ischaemia	234	62	172	0.0003
LGE	295	73	222	<0.0001

## Conclusions

Adenosine stress-CMR is a safe, well-tolerated and feasible investigational modality in high-risk individuals (moderate/severe aortic stenosis, significant LMS stenosis, severe LV systolic dysfunction, asthma/COPD or age >80) with suspected ischaemic heart disease. The incidence of myocardial ischemia or LGE is significantly higher in the high-risk group.

## Funding

This study was funded by the National Institute for Health Research Biomedical Research Unit in Cardiovascular Disease at the University Hospitals Bristol NHS Foundation Trust and the University of Bristol.

